# A primer to traction force microscopy

**DOI:** 10.1016/j.jbc.2022.101867

**Published:** 2022-03-26

**Authors:** Andrea Zancla, Pamela Mozetic, Monica Orsini, Giancarlo Forte, Alberto Rainer

**Affiliations:** 1 Department of Engineering, Università degli Studi Roma Tre, Rome, Italy; 2 Department of Engineering, Università Campus Bio-Medico di Roma, Rome, Italy; 3 Institute of Nanotechnology (NANOTEC), National Research Council, Lecce, Italy; 4 Division of Neuroscience, Institute of Experimental Neurology, San Raffaele Scientific Institute, Milan, Italy; 5 Center for Translational Medicine (CTM), International Clinical Research Center (ICRC), St Anne’s University Hospital, Brno, Czechia

**Keywords:** traction force microscopy, cell adhesion, biophysics, focal adhesion, cytoskeleton, mechanotransduction, mechanosignaling, 2D, two-dimensional, 3D, three-dimensional, BEM, boundary element method, ECM, extracellular matrix, ERISM, elastic resonator interference stress microscopy, FA, focal adhesion, FEM, finite-element method, FTTC, Fourier transform traction cytometry, PA, polyacrylamide, PDMS, polydimethylsiloxane, SIM, structured illumination microscopy, TFM, traction force microscopy

## Abstract

Traction force microscopy (TFM) has emerged as a versatile technique for the measurement of single-cell-generated forces. TFM has gained wide use among mechanobiology laboratories, and several variants of the original methodology have been proposed. However, issues related to the experimental setup and, most importantly, data analysis of cell traction datasets may restrain the adoption of TFM by a wider community. In this review, we summarize the state of the art in TFM-related research, with a focus on the analytical methods underlying data analysis. We aim to provide the reader with a friendly compendium underlying the potential of TFM and emphasizing the methodological framework required for a thorough understanding of experimental data. We also compile a list of data analytics tools freely available to the scientific community for the furtherance of knowledge on this powerful technique.

The premise of mechanobiology is that the mechanical properties of biological tissues can direct given cellular processes, like proliferation, migration, survival, and differentiation. Therefore, mechanobiology entails the understanding of how forces are generated, maintained, and interpreted by cells which actively respond to biophysical stimuli arising from their milieu (Fig. 1).

**Figure 1:**
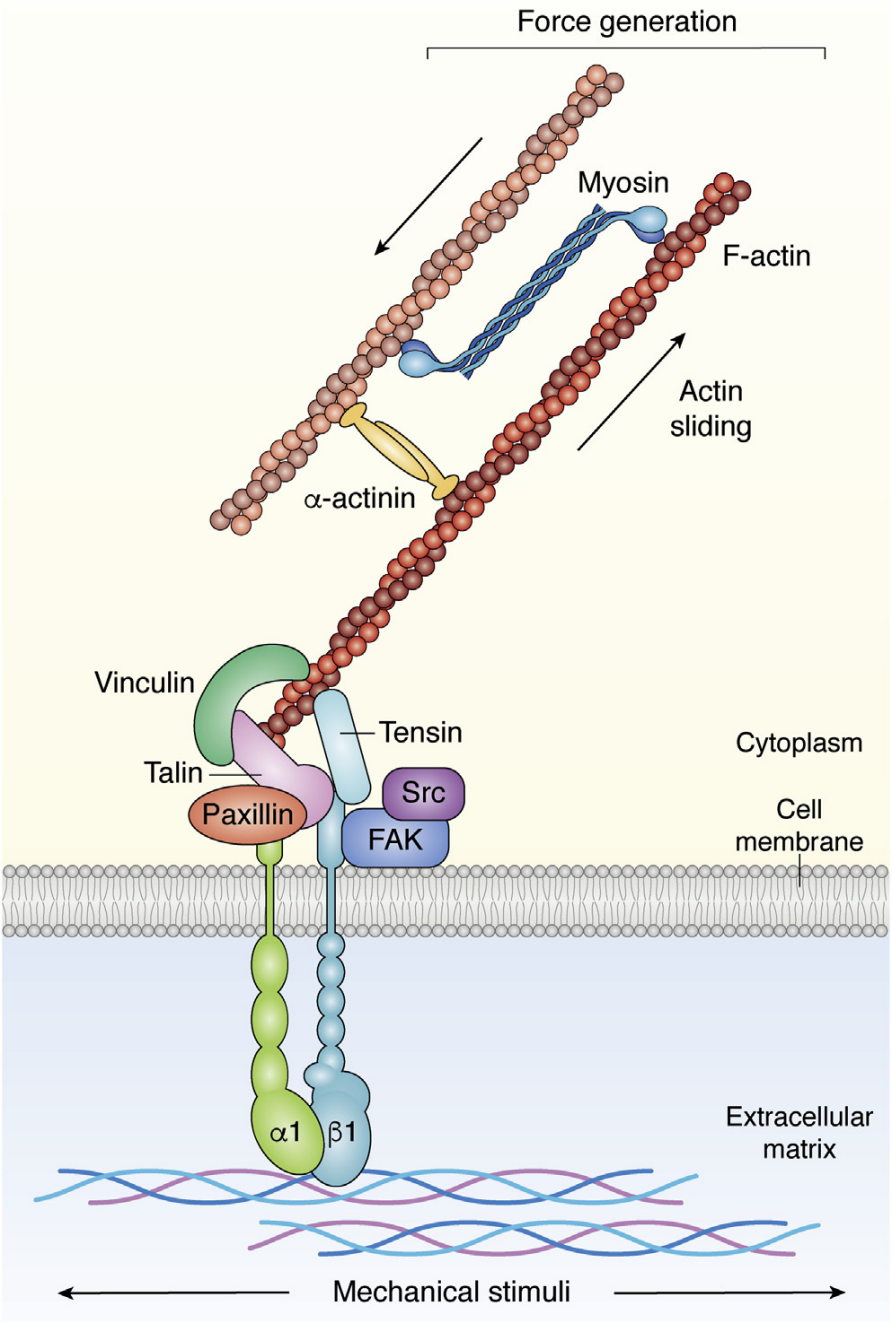
**Simplified schematic representation of the focal adhesions**. Following integrin contact with the extracellular matrix (ECM), docking proteins are recruited that transfer the external stimuli toward the cytoskeleton, where traction forces are generated by the interaction between filamentous actin (F-actin) fibers and the motor protein myosin.

The primary sites of cell interaction to any substrate are the multiprotein complexes which connect the extracellular matrix (ECM) to cell cytoskeleton, the focal adhesions (FAs).

FAs are defined as integrin-based cell-matrix physical contacts transducing and integrating mechanical and biochemical cues arising from the surrounding microenvironment, through the assembly of intracellular multiprotein complexes connected to actin cytoskeleton. The formation of alpha–beta integrin heterodimers and their clustering within the extracellular membrane induces the recruitment of cytoskeleton-docking proteins and the rearrangement of allosterically regulated ones, which in turn start the cellular signaling inward toward the nucleus (1, 2, 3). The proteins that participate in FA formation are distributed in layers. Connected to the integrins, docking proteins like talin, vinculin, zyxin, and tensin are part of the mechanosensing layer. The continuous remodelling of actin cytoskeleton in response to external stimuli is operated by the so-called mechanosignaling proteins, which include paxillin, focal adhesion kinase, Src, and p130Cas, as well as by actin regulators, like Ena-VASP and alpha-actinin, etc. (2). The transmission of the signal inward is ensured by the dynamic rearrangement of the proteins composing the FA complex in response to chemical and mechanical stimuli, thus contributing to both cell–ECM interaction and intracellular signaling (4). Most components of the focal adhesions display some degree of mechanosensitivity (*i.e.*, their localization or conformation changes following the application of physical and biochemical stimuli generated at the ECM). The cooperative activity of these components makes it difficult to determine the specific mechanosensitivity of single FA members. An established example of an FA mechanosensitive protein is talin, a 270-kDa protein which interacts directly with both β-integrin cytoplasmic domain and F-actin. The protein acts as a force buffer by unfolding the numerous rod domains following mechanical load, thus exposing cryptic hydrophobic binding domains able to interact with vinculin (5, 6, 7, 8). The successful binding of vinculin to talin is considered essential to stabilize the interaction between the FA and F-actin and thus transfer the mechanical signal inward (9).

FA dynamics promotes the propagation of forces to the cytoskeleton, as summarized in the study by Spill et al (10). Composed of microfilaments, intermediate filaments, microtubules, and adaptor proteins, the cytoskeleton represents the scaffolding structure of the cell. Its timely rearrangement is necessary for the cell to control its mechanical properties and exert all its functions. A comprehensive description of force transfer from cell periphery toward the cytoskeleton can be found in the review by Martino *et al*. (11).

Due to its ability to directly affect the genetic landscape of the cell in response to extracellular stimuli, modifications in intracellular mechanics induced by cytoskeleton remodeling are now known to also participate in shaping cell identity (12) and have been involved in several pathological processes, including cancer (13).

All this accumulated evidence on the fundamental role of mechanical cues points at the increasing demand for *in vitro* platforms compatible with the measurement of cell–cell and cell–substrate mechanical interactions. Conventional cell culture systems are based on two-dimensional (2D) monolayer cultures routinely used to study cellular mechanisms. However, the predictivity of *in vitro* monolayers when compared to native tissues is known to get poorer with the increase in the system complexity. Moving to three-dimensional (3D) culture allows cells to undergo indirect mechanical stimulation by controlling the rigidity and stiffness of the ECM in which they are embedded (14). In fact, 3D tissue models can be designed to produce and control dynamic mechanical stimuli such as fluid flow, stretch/strain, and compression (15). Quantitative analysis of single-cell behavior easily extends its interpretation and results in higher-scale models such as native tissues, engineered tissue constructs, and organs-on-chip, as reviewed by Ergir *et al*. (16). This experimental landscape is driving the future of computational models in tissue growth and remodeling cases, which are of interest due to their close relationship with the clinical landscape. The field has seen significant advances in recent times, and its development has led to significant improvements in functional tissue engineering approaches (17). Additionally, these new strategies proved to be useful for investigating the molecular basis of cell–cell signaling and contributed to unveil the transmission and regulation mechanisms driving signaling pathways in tissue environments. Special attention has been paid to understand the function and regulation of YAP/TAZ proteins, which are known to play a pivotal role downstream of mechanosensitive Hippo pathway in transducing mechanical signals to the nucleus, in order to dictate focal adhesion assembly, cytoskeleton, and ECM remodeling (18, 19, 20, 21, 22). All these events are crucial to ensure the tight control of cell adhesion, migration, proliferation, and differentiation, which in turn underlies the correct orchestration of vital processes like angiogenesis and immune response, among the others (23, 24).

## Platforms for *in vitro* cell traction measurements

The paradigm shift between the macroscale observation of forces in biomechanics to the single-cell micrometric scale of mechanobiology reasonably began with the work of Harris *et al*. in 1980 (25), in which the first known indirect estimation of cellular traction forces was performed through microscope images. The method was based on cell cultures on distortable sheets of silicone rubber. Although only in a qualitative way, the technique enabled examination of single-cell tractions. Starting from this seminal study, two main approaches have been pursued to study forces at the cellular level: (i) active stimulation methods, which measure cell response to mechanical force application, and (ii) passive methods, which sense mechanical forces generated by cells without applying any external stimulus. Hereafter, we will focus on passive stimulation methods, with particular regard to traction force microscopy (TFM). A more detailed overview on active *versus* passive platforms for single-cell biomechanical characterization can be found in the review by Basoli *et al*. (26).

### Microfabricated platforms

Microfabricated platforms have been investigated to measure cellular tractions in controlled mechanical environments, including both hard silicon-based devices and soft polymer/gel devices. In particular, soft polymer and gel microsystems obtained through soft lithography techniques are characterized by biocompatibility, optical transparency, and the possibility to functionalize the surface as well as to tune its mechanical properties to match those of the *in vivo* environment.

Soft lithography structures are realized by replica molding of a patterned silicon master. Several research groups have highlighted the use of elastomeric microfabricated pillars (microfabricated post-array-detectors) as engineered tools to measure single-cell adhesion forces (27, 28, 29, 30). The analysis of pillar displacement is performed by particle tracking software to detect and label the deflection of each post over the temporal series of images. Tracking can be performed by means of either bright field or fluorescent microscopy (the latter following coating of the pillar tips with fluorescent probes). The lattice arrangement of pillars also offers a means for the calculation of rest (zero-stress) position, which can be obtained by linear fitting starting from the position of the posts not covered by cells belonging to the same row (31). Forces can be calculated with single-pillar resolution from measured deflections, assuming a (quasi) linear relationship between the two entities. A comprehensive discussion on fabrication route, imaging, and evaluation of traction forces can be found in the works of Polacheck & Chen (32) and Gupta *et al*. (33). As a notable advancement in the field, Xiao *et al*. (34) designed a plasmonic micropillar platform with self-organized gold nanospheres, precisely resolving cell tractions across a large field of view. In their work, micropillars were modified with gold nanospheres, which were precisely allocated at the center of each micropillar tip *via* laser annealing process. Gold served as a point source–like light scattering marker, allowing every micropillar to be tracked even under low-magnification objective lenses.

### Traction force microscopy

TFM represents the most widely used technique for measuring cell forces. The core strength of the method is that it can generate quantitative stress maps, resuming the stress of an elastically deformed substrate at the level of the cell adhesion plane. The foundation of the technique is that when a cell is adherent to a soft substrate, it exerts a contractile force causing a strain, which is measurable. TFM commonly relies on thin hydrogel films, endowed with nanoscopic fluorescent beads, which are either embedded in the substrate or attached to its surface to be used as fiducial markers for optical tracking in space and time (35). A typical TFM experiment consists of two subsequent image acquisition phases. During the first phase, the bead positions are recorded in the stressed state when cells are contracting the elastic substrate they have been seeded onto (cell-loaded image). Then, cells are detached by trypsinization, releasing the gel to its unstressed state, where a new image is captured (reference image). The vector displacement field for the substrate at each cell position is computed into a displacement map resuming the deviation (in pixel) of each bead from its reference position as a consequence of the force exerted by the cell (31, 36).

Polyacrylamide (PA) or silicon-based gels are common substrates for TFM. Both types of gels exhibit a linear elastic behavior under deformations produced by cell traction, and their stiffness can be varied over a range of several orders of magnitude. Interestingly, mechanical properties of those gels have been proven not to change under the action of biochemical factors that may occur during a TFM measurement, including cell proteases (37).

## Advanced TFM variants

**Figure 2:**
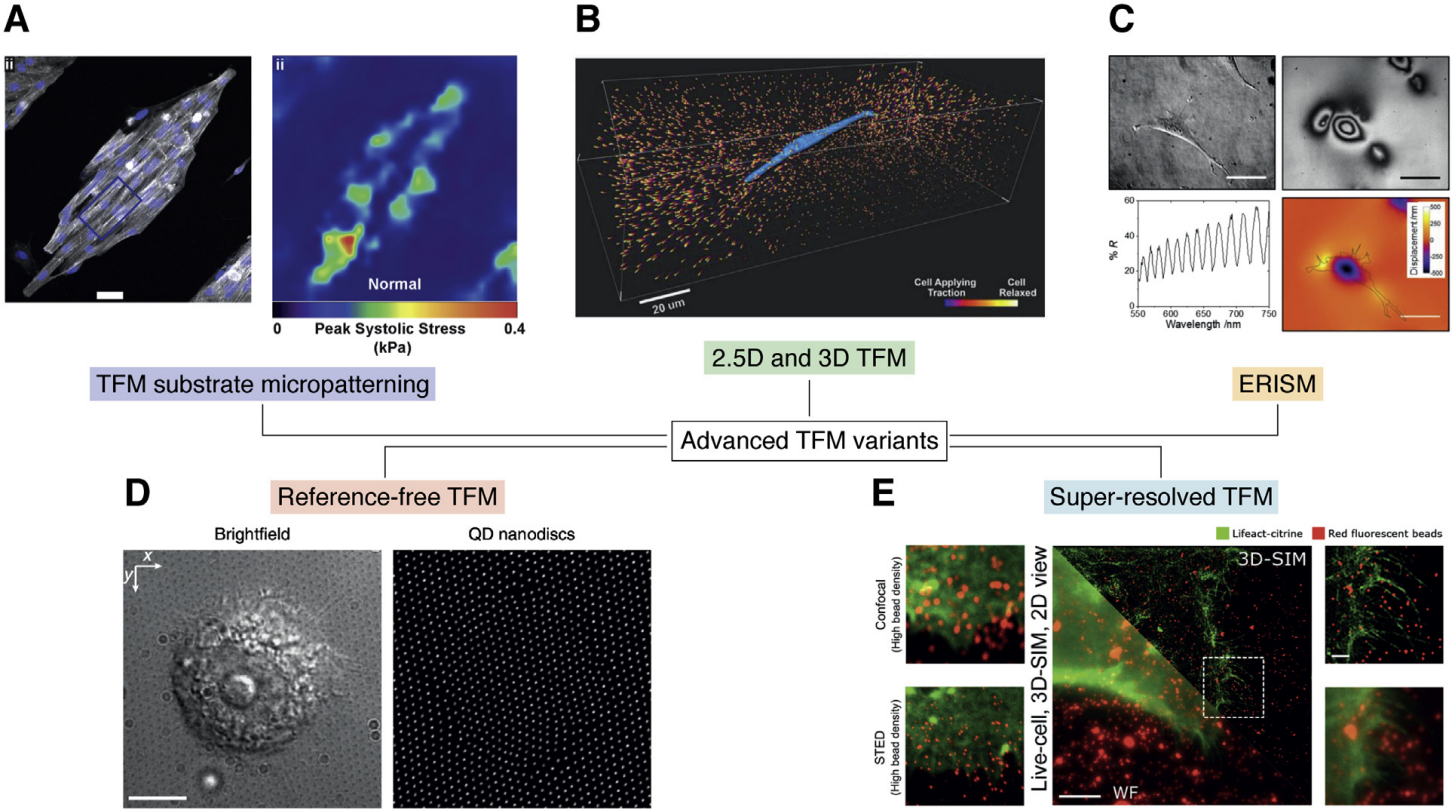
**Advanced variants of TFM technique**. Image thumbnails have been adapted with permission from the corresponding references. *A*, TFM substrate micropatterning (40). *B*, 2.5D and 3D TFM (44). *C*, elastic resonator interference stress microscopy (ERISM) (52). *D*, reference-free TFM (54). *E*, super-resolved TFM (55, 58). QD, quantum dot; SIM, structured illumination microscopy; STED, STimulated Emission Depletion; TFM, traction force microscopy.

The above-described setup for TFM made possible for the technique to reach a high level of diffusion and replicability among different laboratories. Partially accounting for the low throughput of the technique, some groups have introduced dedicated setups, as in the case of Yoshie *et al*., who designed a polydimethylsiloxane (PDMS) contractile force screening platform featuring 96 monolithic independent wells (38). We will not bring further examples of conventional TFM setups (the reader is referred to the comprehensive review by Roca-Cusachs *et al*. for the current state of the art (39)); conversely, we aim to introduce some of its most innovative variants, as outlined in the following subsections and summarized in Figure 2.

### Micropatterning of TFM substrates

Interesting exploitations of surface micropatterning were used to demonstrate alternatives to the more common beads-in-a-gel practice. A novel structure has been realized by Pasqualini *et al*., (40) who applied microcontact printing to the patterned deposition of cell adhesion molecules (fibronectin) on PA gel to direct cell cluster organization. Other scientists produced micropatterned elastomeric substrates by soft lithography by engineering the surface topography with a lattice of either embossed or fluorescent markers (41). The latter approach was achieved by fabricating the pad array with a fluorescent photoresist and by achieving a controlled peel-off of the resist which remained embedded on the PDMS surface.

Overall, micropatterning is an interesting approach which adds an additional degree of control over cell arrangement and which can be used to externally influence cytoskeleton architecture and cellular polarization by tailoring focal adhesion distribution, thus impacting cell migration, growth, and differentiation (42).

### 2.5D and 3D TFM

Location and origin of the normal tractions with respect to the adhesive and cytoskeletal elements of cells can be further modelled to consider the 3D nature of cellular forces acting on planar 2D surfaces (hereby the notation ‘2.5D’). It is worth noting that under elongated focal adhesions, upward and downward normal tractions are more likely to appear on distal (toward the cell edge) and proximal (toward the cell body) ends of adhesions. The resulting rotational moments affect focal adhesions by either protruding or retracting peripheral regions. To measure this, Legant *et al*. (43) developed a 2.5D expansion of the TFM protocol.

Full 3D TFM was designed to measure the traction field of cells that, instead being seeded on top of the substrate, are embedded within an ECM-like 3D environment (44). The measurement steps leading to the displacement field are the same that we already met for standard TFM. From there on, the discrete set of displacement data is converted into a continuous displacement field by means of interpolation. The strain field is then evaluated through a numerical evaluation of the spatial gradient of the above-calculated displacement field. Since the mechanical properties of the hydrogel are known and its constitutive model is defined, the stress field can be calculated without any *a priori* assumption of stress state or ECM geometry. Interestingly, in this case we are not bound to infinite substrate requirements typical of the Boussinesq theory (45, 46) (this topic will be further explained in Box 1). Several significant works have been published on this subject (47, 48, 49, 50), also detailing methods for the numerical solution of the problem (51).

BOX 1 **Mathematical framework**

The math underneath the reconstruction of traction forces in TFM relies on the theory of linear elasticity (96). Substrates are tunable in their characteristics and can be assumed as isotropic, homogeneous, and linear. As such, they are defined by two parameters, namely the linear elastic modulus (Young’s modulus) *E* and the Poisson’s ratio *ν* (97).

TFM problems can be solved directly (*direct TFM*) by calculating the strain field **ε** from the measured displacement field **u**, according to the linearized expression, which holds for small strains:

(1) $$\varepsilon_{i,j}=\frac{1}{2}\left(\frac{\partial u_{i}}{\partial x_{j}}+\frac{\partial u_{j}}{\partial x_{i}}\right)$$

with $u=\;\left(u_{1},u_{2},u_{3}\right)$ and $x=\;\left(x_{1},x_{2},x_{3}\right)$. The stress field σ can then be derived by the constitutive law for linear elasticity (Hooke’s law):

(2) σ=cε

where **c** is the stiffness tensor, which describes the substrate properties (can be expressed in terms of *E* and *v*).

Direct TFM is a relatively recent approach since its implementation demands for high-resolution and high-accuracy measurement of the displacement field, which is mandatory for accurate strain reconstruction.

Most commonly, an *inverse TFM* approach is followed, where cell tractions **t** can be described in terms of the displacement field u using a convolution approach, described by the following Fredholm integral (98), which makes use of the Green’s function **G**:

(3) $$u\left(r\right)=\;\int dr^{\prime }G\left(r-r^{\prime }\right)t\left(r^{\prime }\right)\text{.}$$

**G** describes the impulse response (*i.e.*, the output of a linear system with zero initial conditions and a unit impulse function as the input) of the system to a point load. The integral in Equation 3 can be interpreted as a summation, so that each displacement **u** located at r=(x,y,z) is the net effect of all the traction forces **t** acting in $r^{\prime }=\left(x^{\prime },y^{\prime },z^{\prime }\right)$.

In typical TFM settings, where cell-induced displacements are ca. two orders of magnitude smaller than the substrate thickness (usually in the 50–80 μm range), the Boussinesq approximation of an infinite half-space (*i.e.*, the assumption of infinite thickness) can be applied.

The generic Green’s function is a 3 × 3 tensor. However, given the incompressible nature of TFM hydrogel materials (*ν* ≅ 0.5), decoupling occurs between in-plane tractions and out-of-plane displacements (namely, *G*_*13*_ *= G*_*23*_ = 0). Additionally, in most practical cases, displacement vectors are experimentally measured on the *x-y* plane only, which further reduces the problem to a 2D one, characterized by the following Green’s function (for the infinite half-space):

(4) $$G\left(r\right)=\frac{1+\nu }{\pi Er^{3}}\left[\begin{array}{cc} \left(1-\nu \right)r^{2}+\nu x^{2} & \nu xy\\ \nu xy & \left(1-\nu \right)r^{2}+\nu y^{2} \end{array}\right]$$

with r=|r|. Note that the transformation kernel is a nondiagonal 2 × 2 matrix because tractions in *x* or *y* directions separately induce displacements in both *x* and *y* directions (96). For TFM application, the traction field should be reconstructed from the experimentally measured displacement field. It is therefore necessary to face and solve an inverse problem. In particular, due to the long-ranged (1/r) nature of the Green’s function, such a problem is defined as “ill-posed”, meaning that the solution may be not unique or not continuous with the data. In our settings, reconstructed traction **t** results to be extremely sensitive to any change in **u**. This is particularly daunting when working with experimental data which are intrinsically affected by noise. For this reason, regularization theories have been developed, following the concept of restricting the space of acceptable solutions by choosing the function that minimizes an appropriate functional, as proposed by Tikhonov (99). There are several ways to calculate the traction forces from the displacement field (100, 101). This can be done in real space (*e.g.*, *via* boundary element method [BEM]) or, more commonly, in the spatial frequency domain (Fourier space). All these methods have important methodological considerations, which have been exhaustively addressed in the primary literature and carefully reviewed by Schwarz and Soiné (102). A brief outline is provided in the following.

**Fourier transform traction cytometry**

Butler *et al*. (103) were the first to solve the Fredholm integral for TFM in the Fourier space and introduced the Fourier transform traction cytometry (FTTC) method, which represents by far the most common approach to force reconstruction. FTTC exploits the convolution theorem: the Fourier transform of a convolution of two functions is the product of the Fourier transforms of the two functions. In Fourier space, the convolution integral in Equation 3 factorizes into a product, and traction field can be calculated as follows:

(5) $$\tilde{t}\left(k\right)=\;\tilde{G}{\left(k\right)}^{-1}\tilde{u}\left(k\right)$$

where ‘tilde’ indicates a Fourier transform and **k** is the spatial frequency.

FTTC represents a computationally efficient method for traction reconstruction. However, FTTC is sensitive to experimental noise in the displacement field, which can result in altered reconstructed tractions. For this reason, many researchers have emphasized the need for a regularization strategy in FTTC, seen as a compromise between solution accuracy and the stability. For instance, 0th order Tikhonov regularization can be applied, and Equation 5 can be reformulated to include a regularization term λ:

(6) $$\tilde{t}=\left({\tilde{G}}^{T}\tilde{G}+{\text{λ}}^{2}I\right){\tilde{G}}^{T}\tilde{u}$$

with I the identity matrix.

Several criteria have been proposed for the selection of the optimal regularization parameter. It can be determined in the framework of the Bayesian theory (104) by comparison with a maximum *a posteriori* estimator of the traction, as suggested by Plotnikov *et al*. (100). Alternatively, the selection can be performed graphically from the plot of $log||t|{|}^{2}\;vs.\;log||Gt-u|{|}^{2}$, which appears to have the shape of the letter “L” (hence the term “L-curve criterion”): at the corner of the L-curve, there is the best agreement between regularization and data (105). Other selection criteria occur, many of which have not found an application in TFM yet, as thoroughly reviewed by Huang *et al*. (106).

**Figure 3:**
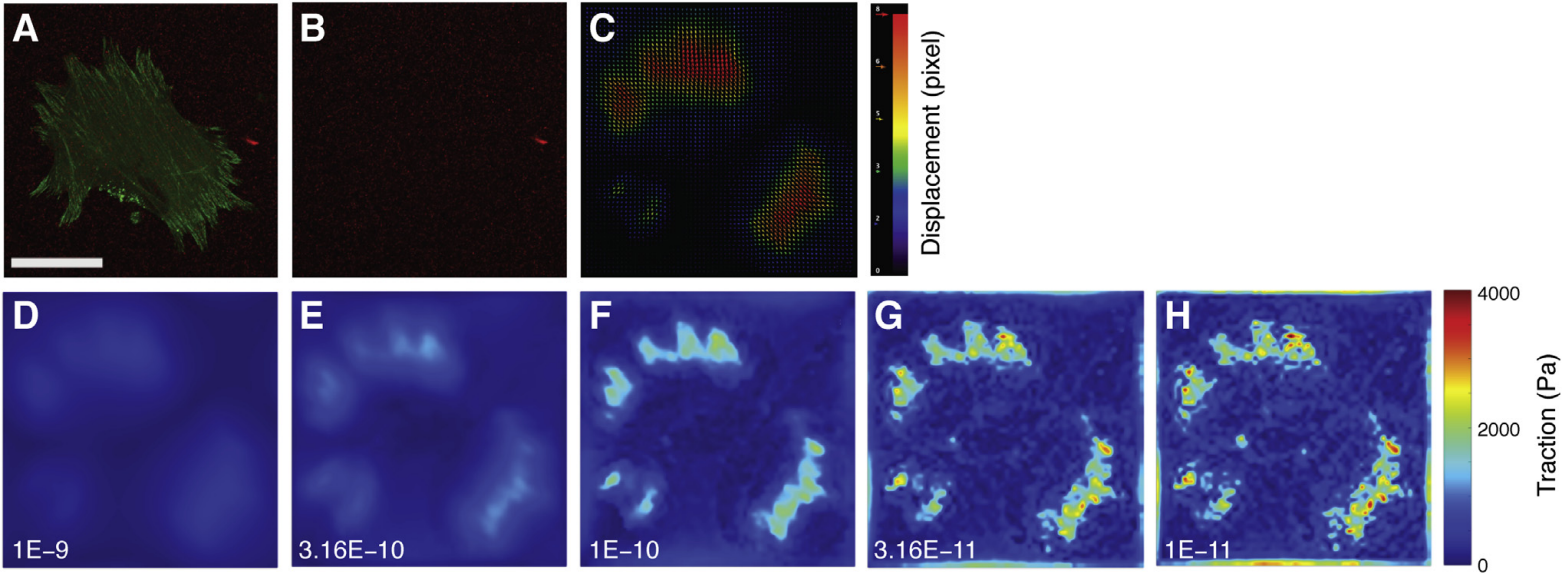
**Effect of the chosen regularization parameter on the estimation of the traction force magnitude**. *A*, sample cell, expressing a fluorescent variant of the FA-associated protein paxillin (in green) and adherent to a 15-kPa (shear modulus) polyacrylamide gel laden with fluorescent nanoparticles (in red). Scale bar: 50 μm. *B*, reference image of the same ROI after cell detachment by trypsinization. Datasets were processed using PIV/FTTC plugins for ImageJ (see BOX 2 for details on software tools), and the resulting traction maps were post-processed in MATLAB (MathWorks, R2019b) for visualization. *C*, displacement vector field calculated by PIV algorithm. *D*-*H*, traction maps obtained from the displacement field in (*C*) using FTTC plugin with different values for the regularization parameter (in the range 1E-9 – 1E-11). The same colormap value ranges were used for all traction maps, emphasizing the impact of the chosen regularization parameter on the resulting solution. FA, focal adhesion; FTTC, Fourier transform traction cytometry; PIV, particle image velocimetry; ROI, range of interest.

From a practical point of view, no consensus has been reached on the application of regularization strategies to experimental data. Many authors recommend a single regularization parameter to be chosen for a given TFM experiment (*i.e.*, for a set of stressed *versus* reference micrograph pairs, acquired in the same experimental conditions), although other studies tune the regularization parameter for each single dataset to account for biological and experimental variability. Undoubtedly, such arbitrariness may introduce systematic errors and makes it hard to compare results in the literature. Figure 3 exemplifies how the choice of the regularization parameter may impact the cell traction reconstruction *via* FTTC.

**Boundary elements method**

BEM is a well-known numerical method for solving integral equations. Application to plane stress problems is known since early 1980s following the work of Telles and Brebbia (107). Indeed, BEM was the first method adopted for the reconstruction of cell traction forces by Dembo *et al*. (108), and the method was further optimized for TFM applications by Sabass *et al*. (109).

The foundation underlying BEM is that the integral in Equation 3 can be discretized on a computation mesh having nodes close enough to justify interpolation. As the name suggests, the computational mesh requires confinement in a boundary, which corresponds at minimum to the cell shape. Nodes are created within the boundary at locations where displacement has been measured, and the domain is discretized by triangulation. Force reconstruction is performed through a regularization scheme, commonly performed *via* 0th order Tikhonov regularization.

Performance of BEM is considered superior to FTTC; however, the computational effort is significantly higher, and the two methods share the tendency to underestimate forces at adhesion sites.

**Finite-Element Method**

Finite-element method (FEM) is a numerical technique used to produce approximate solutions to partial differential equations, as well as integral equations. The goal is to reduce the complexity of the problem to a system of ordinary differential equations that are solved by numerical integration inside a problem-defined domain. The space of the problem is then discretized and subdivided into a finite number of regions (elements) where equations are solved locally (110). FEM has the advantage that it can be adapted to complex geometries and governing equations. For this reason, FEM has found significant use in 3D-TFM, where complex cell boundaries prevent the use of analytical solutions to the elasticity equations in the traction force reconstruction process (111, 112). FEM is also suited to nonlinear material models and geometric nonlinearities resulting from large deformations. A notable example of a FEM-based approach to solve traction forces is found in the work by Kulkarni *et al*. (113), who compared the performance of FEM with regularized FTTC in traction reconstruction on experimental and simulated data.

BOX 2 **Available software tools**

Over the years, several software packages have been released to calculate traction forces and other features of mechanobiological relevance. Here, we present a selection of free tools that could be helpful to the reader.

**TFM in ImageJ**

**Figure 4:**
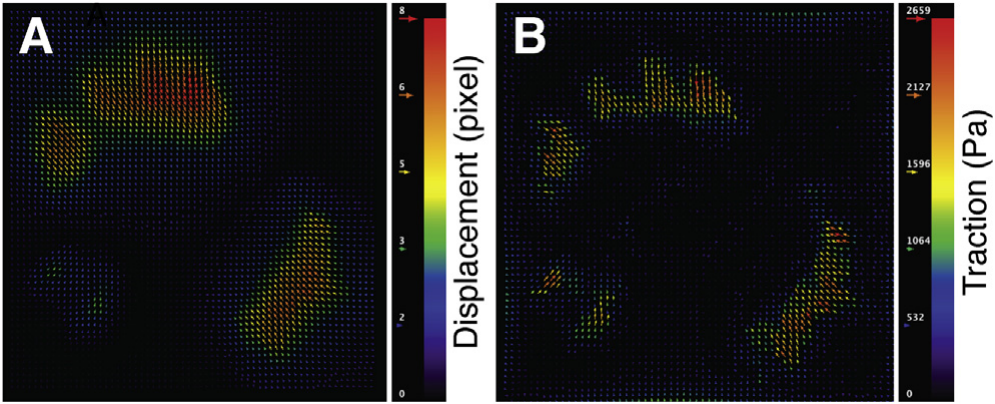
**Output of TFM analysis performed by PIV/FTTC plugins for ImageJ**. Source dataset is as in Figure 3. *A*, vector plot of the displacement field (in pixels). *B*, vector plot of the cell traction field (in Pa). FTTC, Fourier transform traction cytometry; PIV, particle image velocimetry; TFM, traction force microscopy.

A tool for referenced TFM is freely accessible as a set of plugins for ImageJ (114), downloadable from the developer’s website https://github.com/qztseng/imagej_plugins, (Accessed January 21, 2022) (Fig. 4). The tool was developed by Tseng *et al*. (115) starting from the work of Schwarz *et al*. (116). The software package includes three plugins, namely:

1. Template matching: performs alignment between the reference and the “stressed” images compensating for experimental errors (*e.g.*, microscope stage drift).
2. PIV: calculates the displacement field (in pixels) using particle image velocimetry with a user-defined searching window (117);
3. FTTC: calculates the traction map starting from the displacement field, given the substrate-constitutive parameters, the image spatial calibration (pixel/μm), and the regularization parameter λ.

This tool is overall computationally efficient; however, it does not guide the user through the selection of the regularization parameter, nor it allows the user to select the regularization strategy (118).

Further implementation of those ImageJ plugins has been provided by Martiel *et al*. (119). In a synthetic guide, they explained the microscopy and software *how-to* and released a *macro* for automating the calculation procedure and for deriving the mechanical energy stored in the deformed gel.

**TFM in MATLAB**

**Figure 5:**
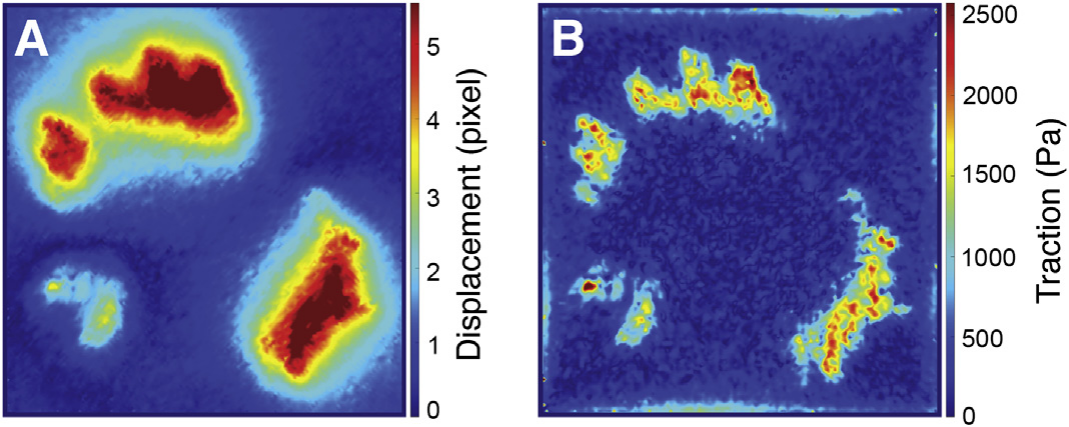
**Output of Danuser Lab TFM package in MATLAB**. Source dataset is as in Figure 3. *A*, displacement map (in pixels). *B*, traction intensity map (in Pa). TFM, traction force microscopy

The group of Prof. Danuser released a MATLAB package to reconstruct traction forces, freely downloadable from GitHub repository https://github.com/DanuserLab/TFM, (Accessed January 21, 2022) (Fig. 5). The software uses a referenced method, quantifying the deformation of the gel by image-based tracking fiducials on the reference substrate and on the deformed one. Traction force reconstruction is accomplished *via* L1-regularization. The tool generates traction magnitude heatmaps as well as traction vector fields. The software also supports L2-based BEM and FTTC algorithms. The elements of novelty of the reconstruction algorithm have been published in the study by Han *et al*. (120).

A multipurpose MATLAB tool for the analysis and solution of ill-posed problems has been released by P. C. Hansen, which can be found in the MATLAB Central repository (under the name ‘regtools’) https://www.mathworks.com/matlabcentral/fileexchange/52-regtools, (Accessed July 26, 2021). Detailed explanation is provided in the book by the same author (121). The package represents a useful resource for the readers interested in developing their own analysis tools.

A MATLAB package for computing 3D TFM in the case of large deformations (LD-3D-TFM) has been developed by the group of Prof. Franck at the University of Wisconsin-Madison and distributed through GitHub repository https://github.com/FranckLab/LD-3D-TFM, (Accessed January 21, 2022). The package includes both the FIDVC algorithm, which is used to calculate 3D deformation fields from micrographs, and the solver to reconstruct force fields from 3D deformations, according to the method published by the same authors (49).

More recently, a comprehensive tool for 4D (x,y,z,t) TFM (TFMLAB) based on an FEM engine has been developed as a MATLAB package by Barrasa-Fano *et al*. (122) and made available on Github repository https://github.com/ElsevierSoftwareX/SOFTX-D-20-00104, (Accessed January 21, 2022). The tool integrates all the computational steps to calculate active cellular forces from confocal microscopy images, including image processing, cell segmentation, image alignment, matrix displacement measurement and force recovery.

**Other software tools**

*SarcTrack* is a MATLAB software program designed by Toepfer *et al*. (123) and available on GitHub https://github.com/HMS-IDAC/SarcTrack2, (Accessed January 21, 2022). The tool determines sarcomere count and any changes in sarcomere length. In return, the algorithm computes sarcomere percent contraction. Cell contraction is obtained from the imaging of labelled z-disc or m-line pairs inside individual sarcomeres.

Koch *et al*. (47) delivered the source code of the tools developed to calculate the strain energy in 3D gels, freely accessible as supplementary information to their article.

### Elastic Resonator Interference Stress Microscopy

In classical TFM, the calculation of the displacement field requires a zero-force reference image. Notably, to get this reference image, cells need to be removed from the substrate and cannot be further analyzed. A novelty in this regard is represented by the elastic resonator interference stress microscopy (ERISM) technique, developed by Kronenberg *et al*. (52). ERISM has the great advantage of being direct and nondestructive. Deformations, instead of being calculated by observing bead displacement or pillar bending, are measured by interferometry. Another great advantage of the technique is that imaging is performed *via* widefield microscopy: multiple cells can be observed simultaneously, and phototoxicity phenomena are almost absent.

In this technique, a soft layer (stiffness ca. 1 kPa) of silicone rubber is used to fabricate a microchamber to be filled with culture media. The optical cavity is sandwiched between two semitransparent layers coated in gold. Cellular forces cause wrinkles on the surface of the substrate which are observed at selected wavelengths using an optical microscope endowed with a tunable monochromatic light source (monochromator). Dark fringes are detected at positions where the actual thickness of the cavity fulfills a resonance condition, and the resulting reflectance at that position is hence attenuated. Analysis of the fringe pattern gives a readout of cell-induced deformation field, with an estimated lateral resolution of ca. 1.6 μm. A detailed discussion on ERISM working principle has been provided by Liehm *et al*. (53).

### Reference-free TFM

Another approach which has been proposed to bypass the acquisition of a reference image is the one of Bergert *et al*. (54). The method is based on electrohydrodynamic nanodrip printing of quantum dot islets (nanodiscs) into confocal monocrystalline arrays on the surface of compliant PDMS substrates with a precision of 30 to 45 nm. Upon imaging of the deformed substrate *via* fluorescence microscopy, the position of each individual nanodisc is determined by calculating its centroid. A triangular mesh is then derived from the nanodisc positions, and displacement is obtained by relaxing the mesh to an equilateral unstressed configuration. From the vector displacement map, surface tractions are calculated on the deformed body by nonlinear finite-element analysis. Moreover, the platform allows for the calculation of out-of-plane forces by recording the normal (vertical) displacement, which is detected by collecting a 3D z-stack of the quantum dot array and performing fluorescence intensity profiling along the normal axis.

### Super-resolved TFM

The adaptation of optical super-resolution microscopy to TFM has led to considerable innovation in the field. Colin-York *et al*. (55) coupled the high spatial resolution of STimulated Emission Depletion microscopy (56) with TFM into super-resolved TFM. The inherent limit of TFM lies in its capability to distinguish fiducials in the stressed substrate, defining its spatial resolution. Indeed, the higher the complexity of the traction field, the higher the required density of fiducial markers. On the other side, if the bead density is too low, traction information is inevitably lost, no matter our ability to resolve individual beads. Here lies the strength of super-resolved TFM, characterized by an increased sampling resolution, with an overall force sampling 5 times better than conventional TFM. More recently, the use of structured illumination microscopy (SIM) (57), a super-resolution technique bringing lateral resolution to 100 nm and axial resolution to 300 nm (almost two-fold better than confocal scanning laser microscopy) has represented a significant advancement in TFM settings (58). Besides, SIM is a widefield technique, inherently fast in acquisition and lower in dose (which is a typical issue in STimulated Emission Depletion). Furthermore, by optimizing the TFM substrate material to match the refraction index of glass, the same group successfully applied total internal reflection fluorescence illumination mode to SIM-based TFM, further pushing the resolution limit along the z axis (59).

## Application of TFM: case studies

In the following, we aim to provide the reader with a (nonexhaustive) set of case studies demonstrating the versatility of TFM for characterizing cell-generated forces in diverse biological scenarios. Paradigmatic examples are summarized in Table 1, with indication of the biological inquiry and the cell model used.

**Table 1:** A list of relevant applications of traction force microscopy, with a detail of the biological inquiry and the cell models used

Biological inquiry	Cell type	Ref
Role of substrate stiffness	Human mesenchymal stromal cells	(66, 67, 68)
	Neonatal rat ventricular myocytes	(40, 69)
	Human embryonic stem cells	(70)
	Human cardiac fibroblasts	(72, 73)
	Cardiomyocytes	(71)
	Epithelial bladder cancer cells (T24)	(93)
	Breast cancer cells (MCF10A/MCF10AT, MDA-MB-231)	(91, 92)
	Lung cancer cells (A-549, BEAS2B)	(91)
	Prostate cancer cells (PC3, PrEC)	(91)
Cell contraction	Human iPSC-derived cardiomyocytes	(124)
	Neonatal rat ventricular myocytes	(40)
	Valve interstitial cells	(74)
	Breast cancer cells (MCF10A/MCF10AT)	(92)
	Epithelial cells	(31)
Cell migration	Murine fibroblast line (NIH/3T3)	(78)
	Goldfish fin fibroblasts (CCL-71)	(80)
	MDCK epithelial cells	(125, 126)
	*Dictyostelium discoideum*	(82)
	Breast cancer cells (MCF10a/MCF10AT, MDA-MB-231)	(91, 92)
	Lung cancer cells (A-549, BEAS2B)	(91)
	Prostate cancer cells (PC3, PrEC)	(91)
Cell invasiveness	Lung cancer cells (A-125, A-549)	(47)
	Breast cancer cells (MDA-MB-123, MCF7)	(47)
	Squamous carcinoma cells (A-431)	(47)
	Epithelial bladder cancer cells (T24, RT112)	(94)
Focal adhesion organization	Human foreskin fibroblasts	(41)
	Goldfish fin fibroblasts (CCL-71)	(80)
	HaCaT keratinocytes	(127)
	Mouse embryonic fibroblasts	(19)
	Breast cancer cells (CAL51)	(12)
	Murine fibroblast line (NIH/3T3)	(22)
	Epithelial ovarian cancer cells (OVCAR5)	(18)
	Breast cancer cells (MDA-MB-231)	(18)
	Prostate cancer cells (PC3)	(18)
	Colorectal adenocarcinoma cells (SW480)	(18)
	Mouse embryonic fibroblasts	(19, 20)
	Breast cancer cells (CAL51)	(12)

### Cardiac biology

The process leading to injury repair in organs exposed to either acute or chronic stresses occurs *via* the deposition of a scar-like ECM, in a phenomenon dubbed as tissue remodeling (60). The negative remodeling of the scar tissue due to excessive ECM deposition usually leads to the establishment of a hostile fibrotic milieu, the loss of tissue compliance, and eventually organ dysfunction (61, 62, 63). The establishment of fibrosis and its detrimental effects on organ function can be observed in the heart, where the phenomenon has been studied extensively (64, 65).

By studying decellularized rat hearts harvested from the fetal, neonatal, and adult development stages, Gershlak *et al*. (66) provided evidence that changes in cardiac ECM chemical composition occur during organogenesis and in adulthood. This phenomenon is paralleled by an increase in ECM stiffness only during the passage between fetal and neonatal life. Decellularized matrices were additionally used in combination with PA gels as substrates for TFM to investigate the ability of human mesenchymal stem cells to generate force as function of different substrate stiffnesses (66, 67, 68).

Jacot et al. investigated the changes observed in neonatal rat ventricular myocytes during their maturation on substrates with controlled stiffness and demonstrated the role of ECM elasticity in dictating the capability of the cell to develop aligned sarcomeres and stress fibers (69). Substrates with an elastic modulus of 10 kPa, which resemble the stiffness of the native myocardium, were shown to favor sarcomere alignment, while stiffer and softer substrates hindered sarcomeric unit formation.

Similar results were obtained by using cardiomyocytes derived from human embryonic stem cells or isolated embryonic cardiomyocytes cultured on flexible substrates. In this case, matrices that mimic the elasticity of the developing myocardial microenvironment were shown to be optimal for transmitting contractile work to the matrix and for promoting actomyosin striation (70). The superior tuneability of PA hydrogels allowed researchers to investigate cardiomyocyte force generation on substrates spanning over a wide range of elastic moduli between 1 and 500 kPa. Findings were applicable to both physiological and pathological conditions of the heart, depicting a shift in cell behavior as a function of substrate stiffness. In fact, the contraction amplitude was found to be stable in cells cultured onto stiffer substrates, while the force level increased to account for the increased stiffness. Interestingly, the authors described an elasticity-independent organization of the cell contractile apparatus (*i.e.*, myofibril organization) (71).

Moving over to the pure quantification of the single-cell response, Pasqualini et al. investigated the relationship between contractile proficiency and metabolism in neonatal rat ventricular myocyte cells, calculating the ratio between the contractile work done by the cells and the metabolic energy provided by the mitochondria. To do so, cells were cultured on gels with stiffness mimicking soft/immature (1 kPa), physiological (13 kPa), and stiff/pathological (90 kPa) cardiac microenvironments, while measuring the strain energy of substrates following cell contraction and ATP production (40). Only on substrates mimicking the physiological stiffness, cells presented an optimal balance between energy production and energy consumption: that is, cells required a minimum amount of ATP to produce maximum contractility levels. On stiffer substrates, ATP levels did not change, but mechanical work was lower (stress levels were comparable to physiological ones, but gel displacement was reduced). Softer substrates, on the contrary, were associated to increased ATP levels, reliably associated to increased ATP demand by an immature sarcomere assembly, while the mechanical work associated to cell contraction was minimum. As a result, physiological stiffness resulted in an energetic efficiency that was 2-fold and 200-fold higher than that of stiff and soft substrates, respectively.

The cells responsible for ECM remodeling in the heart are cardiac fibroblasts, which undergo profound phenotypic alterations in the failing heart (65). Human cardiac fibroblasts were shown to develop a differential cellular response as a function of gel substrate stiffness, as a consequence of stiffness-dependent cell polarization and FA size and morphology (72, 73).

Valve cells are also exposed to a high degree of mechanical stress. In order to understand the response of a cell to traction, valve interstitial cells were challenged with long-term uniaxial or biaxial stretching conditions, and their ability to develop traction force was studied as a form of adaptation (74). The results indicated that the initial cell prestress, as provided by the stimulation with either transforming growth factor-β1 or inhibitor of tension blebbistatin, is a key determinant of the reduction in traction forces experienced by stretched cells. The effect was even magnified in the case of uniform biaxial stretching.

### Cell spreading and migration

The motility of eukaryotic cells is needed for many biological processes, such as embryonic development or tissue repair, as well as for the function of the immune system (75). Many pathologies are associated with the dysregulation of cell migration (76).

The generation of mechanical forces is central for regulating the attachment of cells to a substrate, for cell spreading and migration. Thus, the analysis of cell migration requires understanding the spatial and temporal pattern of cell–cell and cell–substrate mechanical interactions (77).

The first attempt at investigating the contribution of cell-generated stresses to migration was described in mouse NIH3T3 fibroblasts and entailed the acquisition of time-lapse images and shear fields of traction stress at a high spatial and temporal resolution (78). These first TFM experiments demonstrated that forces are generated mainly at the actin-rich leading edge of the cell, where the lamellipodium is formed. On the contrary, cell body and the trailing edge were defined as mechanically passive.

These pioneering studies were the first to demonstrate the existence of a frontal towing mechanism for cell migration in two dimensions, where dynamic traction forces at the leading edge actively pull the cell body forward. This theory was recently questioned by studies adopting nanopillars and displaying enhanced spatial resolution to indicate that the highest displacements are produced by the motile cell in correspondence of the perinuclear regions, rather than on the edges (79).

The ability of the cell to exert force on the ECM during migration has been associated with the formation of the FA complexes. However, recent evidence demonstrated the size and overall FA distribution only partially overlaps with the distribution of traction stress. More importantly, at the leading edge, small dynamic adhesions were shown to transmit strong propulsive tractions, whereas stable mature focal adhesions exerted weaker forces (80). Traction forces could be correlated with other cellular aspects directly affecting the chain effect of cellular mechanics. Lemmon et al. (81), for instance, investigated the correlation between cellular traction forces and fibronectin fibril growth at ECM adhesion sites using a microfabricated post-array platform. The results indicated that fibril orientation during growth is dictated by the applied traction force pattern. Interestingly, these findings also suggest that changes in the spatiotemporal distribution of force occur during cell spreading and are needed for optimal matrix assembly.

TFM technique was adopted in combination with algorithms for 3D tracking to measure the force exerted by *Dictyostelium discoideum* crawling on soft hydrogels (82). By generating dynamic force maps of locomotion, the authors demonstrated that *Dictyostelium* cells exert vertical forces comparable in magnitude to tangential ones during their movement and that such forces are not negligible for the correct localization and quantification of cortical forces. Working on a similar model, Del Alamo et al. (83) found that *Dictyostelium* cells produced much larger contractile forces than needed to overcome the resistance from their environment. They also showed that the temporal evolution of the strain energy exerted by the cells on the substrate was quasi-periodic and could be used to identify the stages of the motility cycle.

### Cancer biology

The alteration of cell mechanics has been described as a reliable prognostic factor in a number of cancers, providing a more accessible clinical indicator than the detection of genetic aberration. Indeed, the invasiveness, proliferative capacity, and survival of cancer cells appear to be tightly connected to their mechanical properties, as determined by intracellular tension, engagement with the ECM, as well as specific cellular adaptations to ECM mechanics and/or its proteolytic remodeling (11, 84, 85, 86). This ability is a key feature of metastatic cells, which produce higher traction forces than nonmetastatic ones (87).

In a self-sustained feedback loop, cancer progression is promoted by genetic changes, altering how the cell responds to the microenvironmental stiffness, geometry, and composition state, while remodeling it in ways that promote cancer cell proliferation and spreading (88). Recent studies on different tumor models, including breast, colorectal, lung, and prostate cancer cells, suggest altered ECM and cell mechanical properties play a role in the metastatic behavior (89, 90, 91, 92).

Therefore, cancer biology represents another key field in which TFM finds an application for the investigation of mechanobiological cues. Ambrosi et al. studied the response of T24 bladder cancer cells to hydrogels at different stiffness levels in the 2- to 10-kPa range (93). On stiff substrates (9.9 kPa), the maximum traction generated was about 200 pN/μm^2^. Of note, cells embedded in this matrix produced filopodia which appeared on the edges and attached to the gel out of the cell contour, fulfilling the role of addressing the direction of motion. On substrates with an intermediate stiffness of 6.3 kPa, cell spreading was found to be markedly decreased, as cells acquired a more oval shape and the maximum traction produced decreased to 140 pN/μm^2^. Cells displayed a marked convex shape when cultured on soft substrates (1.95 kPa), and their maximum traction decreased to nearly 50 pN/μm^2^. The authors also investigated cell migration velocity, which was larger on less rigid gels. During cell migration on planar 2D matrices, cells move due to a cyclic process of polarization, protrusion formation, traction generation, and retraction at their rear end. In this condition, inertial and viscous drag forces are negligible. However, cell migrating in a 3D ECM are required to overcome the steric hindrance of the surrounding environment. For this reason, Koch *et al*. (47) devised an experiment aiming to verify if invasive cancer cells exert higher traction forces than less invasive cell lines. The authors compared the invasion profiles of A-125 and A-549 lung carcinoma with MDA-MB-123 and MCF-7 breast cancer and A-431 squamous carcinoma cells. Invasiveness was estimated by measuring the spatial distribution of cell density 3 days after the cells were seeded on a thick collagen gel. Then, the data were compared with strain energy measurements and related to morphology, which happened to be more spindle-shaped for invasive cells *versus* rounder shape for less invasive ones. The results supported an important role of the directionality of the traction force, rather than the overall traction force magnitude as a descriptor of carcinoma cell invasiveness.

Real-time TFM was, instead, employed to demonstrate that the traction stresses generated by epithelial bladder cancer cells having different degrees of invasiveness (T24 and RT112) can be utilized to predict their motility in substrates with low stiffness (elastic modulus ca. 10 kPa). Consistently, the invasiveness was found to correlate with the interplay between the focal adhesions and the cytoskeleton (94).

Another interesting method was designed to quantify traction forces of cancer cells in highly nonlinear 3D hydrogel networks. The technique exploited a finite-element approach based on a constitutive equation which models the complex mechanical behavior of ECM-like hydrogels (95). Investigating MDA-MB-231 breast carcinoma cells cultured into collagen gels, the authors demonstrated that cell traction forces are independent of collagen concentration (and hence, stiffness level) and that breast cancer cells show a peculiar gliding motion with alternating phases of high and low contractility, elongation, migratory speed, and persistence.

These results highlight the necessity to extend the evaluation of traction forces outside the limits of the cell adhesion plane to understand the overall complexity of cell behavior.

## Conclusions

The ability of living cells to efficiently generate and transfer intracellular forces to the surrounding milieu is crucial for cell adhesion, migration, and maturation. Also, the occurrence of aberrant mechanical signals from the ECM and defects in its perception at the cellular level are now considered of physiological and pathological relevance. Although TFM setup has been standardized and its principles are thoroughly detailed in numerous studies and protocols, the processing of data and the extraction of information from TFM measurements remains somehow operator dependent. The intrinsic ill-posed nature of the mathematical problem and nonuniqueness of its solution mandates for numeric approaches which show some margins of arbitrariness. Nonetheless, thanks to the work of some leading research groups, software tools have been made available, which constitute a solid framework for consistent and reproducible data analysis. This effort is expected to contribute to a wider diffusion of TFM for complementing mechanobiology studies at the single-cell scale.

## Conflict of interest

The authors declare that they have no conflicts of interest with the contents of this article.
